# Population Pharmacogenomics in Croatia: Evaluating the PGx Allele Frequency and the Impact of Treatment Efficiency

**DOI:** 10.3390/ijms241713498

**Published:** 2023-08-31

**Authors:** Vid Matišić, Petar Brlek, Luka Bulić, Vilim Molnar, Marina Dasović, Dragan Primorac

**Affiliations:** 1 St Catherine Specialty Hospital, 10000 Zagreb, Croatia; vid.matisic@svkatarina.hr (V.M.); petar.brlek@svkatarina.hr (P.B.); vilim.molnar@svkatarina.hr (V.M.); 2 School of Medicine, Josip Juraj Strossmayer University of Osijek, 31000 Osijek, Croatia; 3 School of Medicine, University of Zagreb, 10000 Zagreb, Croatia; luka.bulic0302@gmail.com (L.B.); marina.dasovic@gmail.com (M.D.); 4 Medical School, University of Split, 21000 Split, Croatia; 5 Department of Biochemistry & Molecular Biology, The Pennsylvania State University, State College, PA 16802, USA; 6 The Henry C. Lee College of Criminal Justice and Forensic Sciences, University of New Haven, West Haven, CT 06516, USA; 7 Medical School REGIOMED, 96450 Coburg, Germany; 8 Medical School, University of Rijeka, 51000 Rijeka, Croatia; 9 Faculty of Dental Medicine and Health, Josip Juraj Strossmayer University of Osijek, 31000 Osijek, Croatia; 10 Medical School, University of Mostar, 88000 Mostar, Bosnia and Herzegovina; 11 National Forensic Sciences University, Gujarat 382007, India

**Keywords:** pharmacogenomics, allele frequencies, drug-metabolizing enzymes

## Abstract

Background: Adverse drug reactions (ADRs) are a significant cause of mortality, and pharmacogenomics (PGx) offers the potential to optimize therapeutic efficacy while minimizing ADRs. However, there is a lack of data on the Croatian population, highlighting the need for investigating the most common alleles, genotypes, and phenotypes to establish national guidelines for drug use. Methods: A single-center retrospective cross-sectional study was performed to examine the allele, genotype, and phenotype frequencies of drug-metabolizing enzymes, receptors, and other proteins in a random sample of 522 patients from Croatia using a 28-gene PGx panel. Results: Allele frequencies, genotypes, and phenotypes for the investigated genes were determined. No statistically significant differences were found between the Croatian and European populations for most analyzed genes. The most common genotypes observed in the patients resulted in normal metabolism rates. However, some genes showed higher frequencies of altered metabolism rates. Conclusions: This study provides insights into the allele, genotype, and phenotype frequencies of drug-metabolizing enzymes, receptors, and other associated proteins in the Croatian population. The findings contribute to optimizing drug use guidelines, potentially reducing ADRs, and improving therapeutic efficacy. Further research is needed to tailor population-specific interventions based on these findings and their long-term benefits.

## 1. Introduction

Pharmacogenomics (PGx) is a field of research that focuses on genomic information and how it affects individual responses to drugs [1]. The field is constantly expanding as new interactions between certain genes and drugs are discovered [2,3,4]. By doing so, PGx allows the optimization of therapeutic efficacy and minimizes the likelihood of adverse drug reactions (ADRs), which are among the most common causes of death in Western countries [5,6,7]. In 2021, there were 9966 reported ADRs in Croatia, which is 148% more than the year prior, according to the Agency for Medicinal Products and Medical Devices of Croatia (HALMED) [8].

Due to insufficient data on the Croatian population, we have recognized the importance of investigating the most frequent alleles, genotypes, and phenotypes of the population to optimize national guidelines for drug use with the goal of reducing ADRs and increasing therapeutic efficacy. Such guidelines already exist in the USA—the Clinical Pharmacogenetics Implementation Consortium (CPIC), the Netherlands—the Dutch Pharmacogenetics Working Group (DPWG), Canada—the Canadian Pharmacogenomic Network for Drug Safety (CPNDS), and France—the French National Network (Réseau) of Pharmacogenetics (RNPGx) [9,10].

Research efforts have shown that a vast majority of individuals carry single nucleotide polymorphisms (SNPs) that are relevant for drug metabolism; such is also the case in the Republic of Croatia, where it was shown that actionable gene-drug pairs were present in 73.7% of patients at the time of pharmacogenomic testing [11]. It is, therefore, prudent to systematically report the population-specific frequencies of the most relevant SNPs, as it carries the potential benefit of tailoring population-specific interventions that may bring long-term health and economic benefits [12].

This retrospective cross-sectional study aimed to investigate the allele, genotype, and phenotype frequencies of drug-metabolizing enzymes and receptors in a random sample of the Croatian population using a commercially available 28-gene panel. The secondary aim of the study was to compare the observed allele frequencies with the available non-Finnish European population data from the GnomAD database.

## 2. Results

A total of 522 patients were included in the study. The population distribution by category is demonstrated below (Table 1). Regarding ethnicity, the population was predominantly white/caucasian, alongside one American Indian/Alaska native subject and one near/Middle Eastern subject. The distribution by sex demonstrated a higher number of female subjects (58 to 42 ratio). Where age is concerned, 81.8% of the analyzed population was within the 31–80 years-of-age range. A smaller number of subjects (14%) were younger than 31 years of age. Only 4.2% of subjects were 81 years of age or older.

### 2.1. Allele Frequencies

Allele frequencies for the investigated genes are shown in the table below (Table 2). Select alleles were compared with the frequencies in the gnomAD database. No statistically significant discrepancy was found for the *CYP2C* cluster, *COMT*, *NUDT15*, *DRD2*, *OPRM1*, or *F II* genes. Minor discrepancies were found for the *CYP4F2*, *GRIK4*, *HTR2A*, *HTR2C*, *IFNL4*, and *F V* genes. A statistically significant difference in allele frequencies between the Croatian and European populations (gnomAD) was not established.

### 2.2. Genotype and Phenotype Frequencies of Enzyme-Coding Genes

Genotype frequencies and their respective phenotypes for the investigated enzyme-coding genes are shown in the table below (Table 3). The most commonly observed genotypes of the analyzed genes in our patients resulted in normal metabolism rate/no additional gene-drug risk phenotypes. Those were observed in *CYP2B6* (51.5%), the *CYP2C9* (57.9%), *CYP2C* cluster (72%), *CYP3A4* (93.9%), *COMT* (intermediate activity 51.7%), *DPYD* (98.7%), *NUDT15* (99.2%), and *TPMT* (95%). *CYP2D6* genotypes mostly resulted in normal metabolizer phenotype (39.1%), followed by intermediate metabolizer phenotype (28.4%), with poor, rapid, and ultrarapid metabolizer phenotypes observed in 5.4%, 0.8%, and 2.7%, respectively. *CYP1A2* rapid phenotype was observed in 88.5%. The *CYP2C19* phenotype was found to be normal in 35.8%, rapid in 30%, and intermediate in 18%. Poor metabolizer phenotypes were most common in *CYP3A5* (88.1%) and *CYP4F2* (51.1%). *UGT1A1* genotypes were predominantly associated with increased and high risk for ADRs, with frequencies of 45.4% and 16.3%, respectively. The *VKORC1* rs9923231 G/A genotype associated with intermediate activity was present in 50.6% of patients, whereas rs9923231 A/A was present in 18.4% of patients. Combined rs1801133 and rs1801131 genotypes resulted in decreased enzyme activity in 89.1% of patients, with almost half having severely decreased activity phenotypes.

### 2.3. Genotype and Phenotype Frequencies of Receptor-Coding Genes

Genotype frequencies and their respective phenotypes for the investigated enzyme-coding genes are shown in the table above (Table 3). Normal metabolism rates and no additional risk phenotypes were most commonly observed in genes *DRD2* (87%), *GRIK4* (79.7%), *HTR2C* (72.8%), and *OPRM1* (76.4%). The *HTR2A* rs7997012 A/A genotype, which is linked to reduced venlafaxine therapeutic response, was present in 21.1%. *IFNL4* rs12979860 C/C normal response genotype to antiviral efficacy was most common with 47.5%, followed by the rs12979860 C/T reduced response phenotype.

### 2.4. Genotype and Phenotype Frequencies of Uncategorized Genes

Genotype frequencies and their respective phenotypes for the investigated enzyme-coding genes are shown in the table below (Table 3). Normal metabolism rates without additional risk phenotypes were most commonly observed in the genes *HLA-A* (96.4%), *HLA-B* (92.9%), *SLCO1B1* (57.5%), *F II* (96.9%), and *F V* (96.7%). *IFNL4* rs12979860 C/C normal response genotype to antiviral efficacy was most common with 47.5%, followed by the rs12979860 C/T reduced response phenotype.

## 3. Discussion

With the growing implementation of pharmacogenomics worldwide, there are a growing number of studies on population-specific differences in allelic and genotype frequencies of drug-metabolizing enzymes [13,14,15]. Public databases such as GnomAD aggregate this data for future research; however, not all populations are always represented. As a European Caucasian population, the Croatian population did not show major differences in allelic variants when compared to other similar populations in previous studies [16,17]. The results of the present study are in line with these observations. No significant difference was observed between the allele frequencies of the study population and those of the European population for the analyzed alleles that are represented in the database. Regarding the previously published studies that investigated the allele frequencies in the Croatian population, we observed mainly concordant results. However, the previously observed allele frequency for CYP2C19*1 was 85% and for CYP2D6*1 was 76.5%; this was not the case in our patient group, where the wild-type *1 allele frequencies for both enzymes were 59.8% and 37.4%, respectively [16]. This discrepancy suggests greater potential for altered substrate metabolism, as the here detected genotypes coded for normal metabolizing phenotypes in 35.8% of *CYP2C19* and 39.1% of *CYP2D6*. Expectedly, the substrates of *CYP2C19*, proton pump inhibitors, are one of the most commonly prescribed pharmacologic agents in Croatia. It is well established that, when present, genotype information should be considered for therapy guidance [18,19,20]. However, a recent study observed a marked difference in allelic frequencies for CYP2B6*4 (24.3% vs. 9.3% in Europe), VKORC1*2 (40.1% vs. 34.9% in Europe), and CYP2C9*2 (14.7% vs. 12.3% in Europe) [21]. In the present study, this was not the case for CYP2B6*4, which was reported at 2.4%. VKORC1*2 (referred to as rs9923231 A in the present study) was found to be even more frequent at 43.7%, which is closer to the European average.

### 3.1. Antiviral Therapy Considerations

*CYP2B6* is included in the CPIC guidelines for efavirenz genotype-based prescribing, noting that careful dose titration should be performed for poor metabolizers or when certain combinations with the *CYP2C19* phenotype are detected [22]. The observed combined frequencies of poor and poor-to-intermediate metabolizer phenotypes in the observed population were 6.5%; for rapid and ultrarapid metabolizers, the combined frequencies were 4%—those results indicate a considerable number of patients that may require dose adjustment based on genotype alone. When considering the CPIC guideline for efavirenz dosing, the intermediate metabolizer phenotype becomes considerable, as it states that a lower initiating dose should be used compared to normal and rapid phenotypes, with an even lower dose for poor metabolizers [22]. The observed frequency of intermediate metabolizers was 37.4%, suggesting that a third of patients would benefit from this recommendation. *UGT1A1* inhibition is a known side-effect of atazanavir therapy, used in antiretroviral therapy; therefore, it is included in the panel as it is an important metabolizer of bilirubin and other substrates [23]. Our results indicate that the majority of the population has an increased risk of atazanavir-related toxicities due to the *UGT1A1* genotype, as the normal risk *1/*1 genotype was observed only in 38.3%; we also noted a relatively high proportion of the *28/*28 genotype, which is associated with Gilbert syndrome. Another gene important for antiviral therapy, namely the anti-hepatitis C virus, is *IFNL4* [24]. *IFNL4* rs12979860 T variants are associated with a reduced likelihood of a sustained virologic response to peginterferon-containing regimens; this allele was detected in 31.5% of the population, with a normal genotype being present in almost half of the population (47.5%) [25].

### 3.2. NSAID and Opioid Analgetic Considerations

Considering the guideline for non-steroid anti-inflammatory drugs (NSAIDs) recommends lower starting doses for intermediate and poor *CYP2C9* metabolizers, as they have an increased risk of ADRs, it is worth noting that in our population, poor metabolizing phenotype was detected in 2 patients (0.4%), poor to intermediate in 1.9%, and intermediate in 17.4% [26]. This should remind clinicians that, although popular and often available over-the-counter, NSAIDs may still cause ADRs in a considerable proportion of our patients, especially knowing their ATK group had DDD/1000/day of 56.61 in the latest report of the national regulator agency [20].

Opioid analgesics, on the other hand, are mainly metabolized by *CYP2D6,* and their effect is also modulated by *OPRM1* and *COMT*, all of which are analyzed by the panel that was used in our institution and are included in the CPIC guideline for opioid therapy [27,28]. It should be noted that the latest version of the guideline explicitly states the dosing recommendation for codeine, tramadol, and hydrocodone based on the *CYP2D6* phenotype. In contrast, the genotypes of *COMT* and *OPRM1*, even though linked to altered responses to opioid therapy, did not reach the level of evidence for a definite recommendation. Ultrarapid and poor *CYP2D6* metabolizers should avoid using both codeine and tramadol due to a risk of toxicity or a lack of therapeutic response, respectively. *CYP2D6* genotypes in the Croatian population we analyzed show that a combined 14.5% are at risk for either of these adverse events, as they were categorized as either poor, rapid, or ultrarapid metabolizers. This is particularly interesting because tramadol was one of the most prescribed pharmacologic agents for non-inpatient use in the Republic of Croatia in the latest report, with a DDD/1000/day of 12.89.

### 3.3. Warfarin and Coagulation Factor Considerations

CPIC guidelines include recommendations for warfarin dosing, which was considered in the Croatian population in the previous studies, as discussed in the previous section. However, CYP2C9 and VKORC1, which were studied, are not the sole genetic factors for altered clearance. *CYP2C* cluster variant rs12777823 A was represented in 14.9%. Interestingly, this SNP was found to be relevant for warfarin clearance in patients of African descent combined with *CYP2C9*, *CYP4F2*, and *VKORC1* genotypes [29]. This finding shows that similar studies should be performed in other ethnic groups, as the observed allele frequency cannot be overlooked. Although the most commonly observed allele for *CYP4F2* was *1 wild-type allele (69.4%), the resulting phenotypes in our population were predominantly reduced activity (51.1%).

The *VKORC1* wild-type allele was the most commonly observed (56.1%), but like with *CYP4F2*, the most commonly observed phenotype was intermediate activity (50.6%), followed by normal (30.8%) and poor activity (18.4%).

Considering the combined observed frequencies of *CYP2C9*, *CYP2C* cluster, *CYP4F2*, and *VKORC1* phenotype combinations in our population, the results point to a much-needed precaution when prescribing warfarin due to a highly possible gene-drug interaction occurrence altering the patient’s response to warfarin. This has been stressed by previous research from Croatian authors and is now further established by adding the observed frequencies of the *CYP2C* cluster and CYP4F2 gene [21,30]. It is also worth noting that ethnic differences were previously observed for *CYP2C9*, *CYP4F2*, and *VKORC1,* suggesting it may be beneficial to add *CYP4F2* testing to algorithms for genotype-based warfarin dosing [31]. This may be true for our population due to the relatively high percentage with reduced activity *CYP4F2* phenotype.

A relatively low-prevalence finding was the variant in *F II* and *F V*, which was found in only 3% of both genes. Both of those genes are included in the panel, as their variants predispose patients to hypercoagulability, which increases the risk of thrombosis for patients on hormone contraceptives.

### 3.4. Fluoropyrimidine and Thiopurine Considerations with Respect to DPYD, NUDT15 and TPMT

Dihydropyrimidine dehydrogenase (DPYD) polymorphisms are a potential cause of severe adverse drug reactions in oncologic patients undergoing fluoropyrimidine therapy [32]. The *DPYD* wild-type allele was the predominantly detected variant (99%) in the present study. This number is higher than what is considered average for European populations [32].

Thiopurine toxicity can be reduced with proactive *NUDT15* and *TPMT* screening, as stated in the respective CPIC guidelines [33]. Both of these genes were predominately of normal-risk phenotype (95% for TPMT and 99.2% for NUDT15) in the analyzed population.

### 3.5. CYP3A Family and HLA

The CYP3A family is one of the most important families of cytochrome P-450 enzymes considering its presence in the liver and intestine, with CYP3A4 and CYP3A5 being the most prominent members [34]. The vast majority of the analyzed population was carrying the wild-type *1 allele for *CYP3A4* (93.9%), whereas the most commonly observed allele for *CYP3A5* was *3 (93.9%), resulting in decreased enzymatic activity. This observation is in line with other Caucasian populations, where the allele frequencies were determined to be between 82 and 95%, as noted by the PharmGKB [35]. *HLA-A* and *HLA-B* polymorphisms may prone patients to adverse drug reactions with carbamazepine, oxcarbazepine, abacavir, and allopurinol. In this study, the increased risk variants were present in 3.6% of *HLA-A* and 7.1% of *HLA-B*, with 3.3% of the patients being positive for increased allopurinol risk [36].

### 3.6. Statin Therapy Implications

As an important hepatic transporter protein, SLCO1B1 activity is responsible for the metabolization of many drugs [37]. Most importantly, according to the available guidelines, statins. Decreased transporter activity due to SNPs in *SLCO1B1* is recognized as an important factor in statin-related ADRs, namely myopathy [38]. Also, CYP2C9 intermediate and poor metabolizers should initiate fluvastatin therapy at lower starting doses. Normal activity of SLCO1B1 was observed in 41.4% of the patients, which is highly important as it puts over half of the patients in the at-risk group who would benefit from proactive testing prior to the start of statin therapy. Moreover, knowing that statins are included in the first and second lines of prevention for major coronary events, they are one of the most commonly prescribed medications, with 58 DDD/1000/day for atorvastatin and 35.02 DDD/1000/day for rosuvastatin in the Republic of Croatia [20]. A recent randomized controlled trial demonstrated the superiority of genotype-guided statin dosing to usual care with an increase in statin initiation and lower LDL cholesterol, further reinforcing the implementation of proactive pharmacogenomic testing in the setting of cardiovascular disease [39].

### 3.7. Clopidogrel Therapy Implications

Another finding of the present study that merits further attention regarding cardiovascular drugs is the prevalence of polymorphisms in CYP2C19 that interact with clopidogrel metabolism. Patients who are poor metabolizers are at risk of decreased therapeutic response as the concentration of their active metabolite is lower. This, in turn, puts the patients at risk of a lack of therapeutic response [40]. In the present study, the CYP2C19 poor metabolizer phenotype was present only in 2.1%, while the rapid and ultrarapid metabolizer phenotypes were present in 30% and 6.9%, respectively. Furthermore, knowing that adherence to the guideline on genotype-based dosing for clopidogrel was found to be non-inferior to the standard approach, patients in our population could benefit from CYP2C19 testing [41].

### 3.8. Beta-Blocker Implications

Beta-blockers were found to be one of the most commonly used drugs in the therapy of patients who reported for pharmacogenetic counseling in our previous study [11]. The DPWG guideline includes recommendations for metoprolol dosing based on genotype, where a decrease in dose and titration are recommended for both intermediate and poor metabolizers [42]. It should be noted, from a clinical perspective, that other beta-blockers such as carvedilol, propranolol, nebivolol, and timolol are all metabolized to a lesser extent by CYP2D6, but no genotype-based dosing guideline is provided by CPIC or DPWG [43]. A recent study demonstrated a statistically significant difference in the incidence of bradycardia in poor metabolizers compared to normal metabolizers and phenoconverted patients [44]. In the present study, intermediate metabolizer phenotypes were present in 25.8% and poor metabolizer phenotypes in 11.1%.

These results show a significant number of patients at risk for suboptimal drug therapy considering cardiovascular medications; therefore, pharmacogenetic testing should be considered for the Croatian population, especially in cases of secondary prevention, where the majority of the abovementioned drugs find their use.

### 3.9. Selective Serotonin Reuptake Inhibitors (SSRI) and Tricyclic Antidepressants (TCA) Implications

Treatment outcomes of SSRI and TCA therapy are greatly influenced by polymorphisms in multiple genes [4]. The gene-drug interactions are influenced by polymorphisms in CYP2D6 (paroxetine, fluvoxamine, venlafaxine, vortioxetine, and both tertiary and secondary amine TCA), CYP2C19 (citalopram, escitalopram, sertraline, and tertiary amine TCA), and CYP2B6 (sertraline), as pointed out in CPIC guidelines [19,45]. As previously stated for other groups of medications, the combined poor, rapid, and ultrarapid *CYP2D6* metabolizer phenotype was present in 14.5% of patients, whereas the combined poor, rapid, and ultrarapid *CYP2C19* and *CYP2B6* phenotypes were present in 39% and 10.5%, respectively.

Although included in the panel, *HTR2A* and *SLC6A4* polymorphisms did not reach a high enough level of clinical evidence to be included in the SSRI guideline. It should be noted that if future guidelines include these genes in their recommendations. 

The high combined variability of detected metabolizing phenotypes should stand to support proactive testing for psychiatric patients, as they are at an increased risk of both ADRs and a lack of therapeutic response, which too often leads to a trial-and-error prescribing approach in the clinical setting. Furthermore, a meta-analysis showed better therapeutic outcomes for patients with major depressive disorder when utilizing genotype-guided prescribing [46].

The main strength of this study is the scope of the analyzed SNPs in 28 genes. In the current study, the comparison with the European population averages from GnomAD revealed no significant differences, indicating a common guideline/protocol may be implemented with our population. We believe this data may be used as a reference point for future studies on allelic and genotype frequencies of drug-metabolizing enzymes, especially if the Croatian population is studied.

The limitations of the present study include the focus on specific SNPs. Although comprehensive, the panel we used targeted loci with a proven gene-drug effect, possibly missing those not included in the panel. This approach, however, has the added benefit that each of the analyzed loci is causal and leads to a change in drug metabolism. Another limitation is based on the retrospective nature of the study protocol. The patients were not asked to identify with respect to their nationality or relatedness at any point during their outpatient hospital visit; therefore, a minor but possible confounder in the dataset includes patients of other nationalities and patients related to one another.

We hope that the results of this study will not only add data for a specific Caucasian population but also serve as a stepping stone for a broader application of pharmacogenomics in Croatian healthcare.

## 4. Materials and Methods

### 4.1. Target Population and Genes

The analysis of the target genes was performed by OneOme, LLC. The isolated DNA was analyzed using PCR probe-based methods to discover potential variant locations. Haplotypes, or inherited variants, are designated based on the legacy nomenclature. The target genes were: *CYP1A2*, *CYP2B6*, *CYP2C9*, *CYP2C19*, *CYP2C* cluster, *CYP2D6*, *CYP3A4*, *CYP3A5*, *CYP4F2*, *COMT*, *DPYD*, *DRD2*, *GRIK4*, *HLA-A*, *HLA-B*, *HTR2A*, *HTR2C*, *IFNL4*, *NUDT15*, *OPRM1*, *SLC6A4*, *SLCO1B1*, *TPMT*, *UGT1A1*, *VKORC1*, *F II*, *F V*, and *MTHFR*.

In the case of the *CYP2D6* gene, the test can detect deletions, duplications/multiplications, and hybrid alleles, but it cannot differentiate duplications that are coupled with deletions. Variants are detected with an accuracy of >99.9%. PCR interference due to reaction inhibitors or compromised DNA quality can occur, but typically produces negative results rather than false-positive results. Results can be inaccurate in cases of non-autologous blood transfusions and organ transplant therapies. Finally, results can extremely rarely be impacted by laboratory errors.

This study retrospectively analyzed the results of DNA samples from 522 patients from a single center in Croatia using the described method. The included patients reported to St. Catherine Specialty Hospital for pharmacogenetic counseling from January 2018 until March 2023. Patients of both sexes and of all age groups were included in the study. No exclusion criterion was used with respect to the patient’s race. The patients’ data was accessed retrospectively through the hospital records system for all the patients. A database was created from the findings, showing each patient’s alleles for each gene. Allele frequency for each gene was determined by dividing the total count of that allele by the total number of alleles in the patient pool for the respective gene. Genotype frequency was determined for each gene by dividing the total count of each unique allele combination by the total number of genotypes in the patient pool for the respective gene. Finally, each genotype was associated with its phenotype in the context of drug metabolization, in accordance with the findings. The genotypes were then grouped by phenotype, and phenotype frequency was determined by dividing the total count of each unique phenotype by the total number of phenotypes.

### 4.2. Comparison with the GnomAD Database

Comparison of our data with data for the general European population was done using the gnomAD database (https://gnomad.broadinstitute.org/, accessed on 4 June 2023). GnomAD is an online resource used for the large-scale gathering of exome and genome sequencing data. For comparison, we used the v2.1.1 data set, which is in line with the GRCh37 reference human genome. The comparison was done only for pharmacogenetic genotypes determined by a single variant. The selected population, as relevant to our population, was European (non-Finnish). The relevant extracted data were the total number of sequences, the number of sequences in which the variant was discovered, and the frequency of the variant. The frequencies were then compared to the corresponding frequencies provided by our data set.

### 4.3. Statistical Analysis

We used the Hardy-Weinberg equilibrium for the initial genotype data quality check. The statistical analysis of the obtained data was performed using the software package IBM SPSS Statistics 24.0 (SPSS, Chicago, IL, USA). The normality of the distribution of individual parameters was tested using the Kolmogorov–Smirnov test of normality. The Chi-square test and Fisher’s exact test were used to assess whether a statistically significant difference in allele frequencies exists between the Croatian and European populations.

## Figures and Tables

**Table 1:** Distribution of subjects by sex, age and ethnicity.

Category	Number	Percentage (%)
**ETHNICITY**		
White or Caucasian	520	99.6
American Indian or Alaska Native	1	0.2
Near/Middle Eastern	1	0.2
**SEX**		
Male	217	41.6
Female	305	58.4
**AGE**		
1–10	11	2.1
11–20	23	4.4
21–30	39	7.5
31–40	78	14.9
41–50	81	15.5
51–60	98	18.8
61–70	87	16.7
71–80	83	15.9
81–90	21	4.0
91–100	1	0.2

**Table 2:** Allele numbers and frequencies in comparison to available gnomAD database data.

	Gene	Allele	Allele Number	Frequency	GnomAD Frequency *
**ENZYMES**	CYP1A2	*1A	323	0.309	
		*1F	649	0.622	
		*1D	11	0.011	
		*1V	15	0.014	
		*1L	18	0.017	
		*1K	6	0.006	
		*1W	16	0.015	
		*1J	6	0.006	
	CYP2B6	*1	644	0.617	
		*4	25	0.024	
		*5	121	0.116	
		*6	254	0.245	
	CYP2C9	*1	803	0.769	
		*2	142	0.136	
		*3	96	0.092	
		*11	3	0.003	
	CYP2C19	*1	624	0.598	
		*2	154	0.148	
		*17	266	0.255	
	CYP2C cluster	rs12777823 G	888	0.851	0.8540
		rs12777823 A	156	0.149	0.1460
	CYP2D6	*1	390	0.374	
		*1x2	9	0.009	
		*2	1	0.001	
		*2x2	2	0.002	
		*2 + *13	1	0.001	
		*2A	163	0.156	
		*2Ax2	12	0.011	
		*2A + *13	6	0.006	
		*3	13	0.012	
		*4	100	0.096	
		*4x2	3	0.003	
		*4 + *4N	9	0.009	
		*4 + *68	61	0.058	
		*4 + *68xN	2	0.002	
		*5	25	0.024	
		*6	7	0.007	
		*9	21	0.020	
		*10	16	0.015	
		*13	3	0.003	
		*13 + *2A	1	0.001	
		*14	1	0.001	
		*35	73	0.070	
		*39	2	0.002	
		*41	110	0.105	
		*41x2	1	0.001	
		*41x3	1	0.001	
		*59	11	0.011	
	CYP3A4	*1	980	0.939	
		*1B	31	0.030	
		*22	33	0.032	
	CYP3A5	*1	63	0.060	
		*3	980	0.939	
		*7	1	0.001	
	CYP4F2	*1	725	0.694	0.7134
		*3	319	0.306	0.2866
	COMT	rs4680 G	502	0.481	0.4802
		rs4680 A	542	0.519	0.5198
	DPYD	*1	1034	0.990	
		*2A	4	0.004	
		rs67376798 T	3	0.003	
		rs67376798 A	3	0.003	
	NUDT15	rs116855232 C	1040	0.996	0.99649
		rs116855232 T	4	0.004	0.003510
	TPMT	*1	1018	0.975	
		*3A	24	0.023	
		*3C	2	0.002	
	UGT1A1	*1	637	0.610	
		*6	4	0.004	
		*28	403	0.386	
	VKORC1	rs9923231 G	586	0.561	
		rs9923231 A	456	0.437	
		rs9923231 G/G resistance allele	2	0.002	
	MTHFR	rs1801133 C	674	0.658	
		rs1801133 T	350	0.342	
		rs1801131 A	692	0.676	
		rs1801131 C	332	0.324	
**RECEPTORS**	DRD2	rs1799978 A	976	0.935	0.93961
		rs1799978 G	68	0.065	0.06039
	GRIK4	rs1954787 T	473	0.453	0.4467
		rs1954787 C	571	0.547	0.5533
	HTR2A	rs7997012 A	485	0.465	0.4546
		rs7997012 G	559	0.535	0.5454
	HTR2C	rs3813929 C	848	0.812	0.8294
		rs3813929 T	196	0.188	0.1706
	OPRM1	rs1799971 A	914	0.875	0.8743
		rs1799971 G	130	0.125	0.1257
**OTHER**	HLA-A	Negative	503	0.964	
		Positive *31:01	19	0.036	
	HLA-B	Negative	485	0.929	
		Positive *57:01	20	0.038	
		Positive *58:01	17	0.033	
	IFNL4	rs12979860 C	715	0.685	0.6793
		rs12979860 T	329	0.315	0.3207
	SLC6A4	La	544	0.541	
		Lg	70	0.070	
		Sa	392	0.390	
	SLCO1B1	*1	17	0.013	
		*1A	443	0.348	
		*1B	427	0.335	
		*5	144	0.113	
		*15	127	0.100	
		*17	40	0.031	
		*21	76	0.060	
	F II	rs1799963 G	1028	0.985	0.98755
		rs1799963 A	16	0.015	0.01245
	F V	rs6025 G	1027	0.984	0.9704
		rs6025 A	17	0.016	0.0296

For the European population (non-Finnish).

**Table 3:** Genotype numbers and frequencies with respective phenotypes.

	Gene	Genotype	Genotype Number	Frequency	Phenotype
**ENZYMES**	CYP1A2	*1A/*1F	206	0.395	rapid
		*1F/*1F	202	0.387	rapid
		*1A/*1A	54	0.103	normal
		*1F/*1W	16	0.031	rapid
		*1A/*1L	9	0.017	rapid
		*1F/*1V	9	0.017	rapid
		*1F/*1L	9	0.017	rapid
		*1D/*1J	6	0.011	rapid
		*1K/*1V	6	0.011	intermediate to normal
		*1D/*1F	5	0.010	rapid
	CYP2B6	*1/*1	189	0.362	normal
		*1/*6	169	0.324	intermediate
		*1/*5	80	0.153	normal
		*6/*6	28	0.054	poor to intermediate
		*5/*6	26	0.050	intermediate
		*1/*4	17	0.033	rapid
		*5/*5	6	0.011	poor
		*4/*5	3	0.006	rapid
		*4/*6	3	0.006	intermediate to normal
		*4/*4	1	0.002	ultrarapid
	CYP2C9	*1/*1	302	0.579	normal
		*1/*2	114	0.218	intermediate to normal
		*1/*3	82	0.157	intermediate
		*2/*3	10	0.019	poor to intermediate
		*2/*2	9	0.017	intermediate
		*1/*11	3	0.006	intermediate to normal
		*3/*3	2	0.004	poor
	CYP2C19	*1/*1	187	0.358	normal
		*1/*17	156	0.300	rapid
		*1/*2	94	0.180	intermediate
		*2/*17	38	0.073	intermediate to normal
		*17/*17	36	0.069	ultrarapid
		*2/*2	11	0.021	poor
	CYP2C cluster	rs12777823 G/G	376	0.720	normal
		rs12777823 G/A	136	0.261	variant present
		rs12777823 A/A	10	0.019	variant present
	CYP2D6	*1/*1	77	0.148	normal
		*1/*2A	57	0.109	normal
		*1/*41	41	0.079	intermediate to normal
		*1/*35	32	0.061	normal
		*1/*4	30	0.057	intermediate
		*1/*4 + *68	24	0.046	intermediate
		*2A/*41	19	0.036	intermediate to normal
		*2A/*4	17	0.033	intermediate
		*2A/*2A	14	0.027	normal
		*2A/*4 + *68	11	0.021	intermediate
		*2A/*35	11	0.021	normal
		*1/*9	10	0.019	intermediate to normal
		*4/*41	10	0.019	poor to intermediate
		*4/*4 + *68	8	0.015	poor
		*1/*3	7	0.013	intermediate
		*4 + *68/*35	7	0.013	intermediate
		*41/*41	7	0.013	intermediate
		*1/*10	6	0.011	intermediate to normal
		*2A/*5	6	0.011	intermediate
		*4/*4	6	0.011	poor
		*4/*35	6	0.011	intermediate
		*35/*41	6	0.011	intermediate to normal
		*1/*5	5	0.010	intermediate
		*1/*59	5	0.010	intermediate to normal
		*4/*10	5	0.010	poor to intermediate
		*4 + *68/*41	5	0.010	poor to intermediate
		*1/*2A + *13	4	0.008	normal
		*1/*6	4	0.008	intermediate
		*9/*35	4	0.008	intermediate to normal
		*1/*2Ax2	3	0.006	ultrarapid
		*1/*4 + *4N	3	0.006	intermediate
		*2A/*2Ax2	3	0.006	ultrarapid
		*4/*5	3	0.006	poor
		*5/*41	3	0.006	poor to intermediate
		*10/*41	3	0.006	intermediate
		*1/*1x2	2	0.004	ultrarapid
		*1x2/*2A	2	0.004	ultrarapid
		*1x2/*2Ax2	2	0.004	ultrarapid
		*1x2/*41	2	0.004	rapid
		*2A/*6	2	0.004	intermediate
		*2A/*10	2	0.004	normal
		*3/*4	2	0.004	poor
		*4/*9	2	0.004	poor to intermediate
		*4 + *4N/*35	2	0.004	intermediate
		*4 + *68/*5	2	0.004	poor
		*5/*5	2	0.004	poor
		*9/*41	2	0.004	intermediate
		*13/*39	2	0.004	intermediate
		*1/*2x2	1	0.002	ultrarapid
		*1/*14	1	0.002	intermediate
		*1/*41x3	1	0.002	rapid
		*1x2/*4	1	0.002	normal
		*2/*4	1	0.002	intermediate
		*2x2/*41	1	0.002	rapid
		*2 + *13/*4	1	0.002	intermediate
		*2A/*4x2	1	0.002	intermediate
		*2A/*4 + *4N	1	0.002	intermediate
		*2A/*9	1	0.002	intermediate to normal
		*2A/*13 + *2A	1	0.002	normal
		*2A/*59	1	0.002	intermediate to normal
		*2Ax2/*4	1	0.002	normal
		*2Ax2/*4 + *68	1	0.002	normal
		*2Ax2/*4 + *68xN	1	0.002	normal
		*2Ax2/*41	1	0.002	ultrarapid
		*2A + *13/*35	1	0.002	normal
		*2A + *13/*41	1	0.002	intermediate to normal
		*3/*4 + *68	1	0.002	poor
		*3/*4 + *68xN	1	0.002	poor
		*3/*5	1	0.002	poor
		*3/*35	1	0.002	intermediate
		*4/*59	1	0.002	poor to intermediate
		*4x2/*4 + *4N	1	0.002	poor
		*4x2/*35	1	0.002	intermediate
		*4 + *4N/*9	1	0.002	poor to intermediate
		*4 + *4N/*41	1	0.002	poor to intermediate
		*4 + *68/*4 + *68	1	0.002	poor
		*5/*35	1	0.002	intermediate
		*6/*41	1	0.002	intermediate
		*9/*13	1	0.002	poor to intermediate
		*35/*59	1	0.002	normal
		*41x2/*59	1	0.002	intermediate to normal
		*59/*59	1	0.002	intermediate
	CYP3A4	*1/*1	459	0.879	normal
		*1/*1B	31	0.059	normal
		*1/*22	31	0.059	intermediate to normal
		*22/*22	1	0.002	intermediate
	CYP3A5	*3/*3	459	0.879	poor
		*1/*3	61	0.117	intermediate
		*1/*1	1	0.002	normal
	CYP4F2	*1/*1	255	0.489	normal
		*1/*3	215	0.412	reduced activity
		*3/*3	52	0.010	reduced activity
	COMT	rs4680 G/A	270	0.517	intermediate activity
		rs4680 A/A	136	0.261	low activity
		rs4680 G/G	116	0.222	high activity
	DPYD	*1/*1	515	0.987	normal risk
		*1/*2A	4	0.008	increased risk (DPD score = 1)
		rs67376798 T/A	3	0.006	increased risk (DPD score = 1,5)
	NUDT15	rs116855232 C/C	518	0.992	normal metabolizer
		rs116855232 C/T	4	0.008	increased risk
	TPMT	*1/*1	496	0.950	normal risk
		*1/*3A	24	0.046	increased risk
		*1/*3C	2	0.004	increased risk
	UGT1A1	*1/*1	200	0.383	normal risk
		*1/*6	2	0.004	increased risk
		*1/*28	235	0.450	increased risk
		*6/*28	2	0.004	high risk
		*28/*28	83	0.159	high risk
	VKORC1	rs9923231 A/A	96	0.184	low activity
		rs9923231 G/A	264	0.506	intermediate activity
		rs9923231 G/G	161	0.308	normal activity
		rs9923231 G/G resistance allele	1	0.002	resistance allele(s)
	MTHFR	rs1801133 C/C– rs1801131 A/A	56	0.109	normal activity
		rs1801133 C/C– rs1801131 A/C	109	0.213	decreased activity
		rs1801133 C/C– rs1801131 C/C	56	0.109	severely decreased activity
		rs1801133 C/T– rs1801131 A/A	121	0.236	decreased activity
		rs1801133 C/T–rs1801131 A/C	111	0.217	severely decreased activity
		rs1801133 T/T– rs1801131 A/A	59	0.115	severely decreased activity
**RECEPTORS**	DRD2	rs1799978 A/A	454	0.870	normal response
		rs1799978 A/G	68	0.130	reduced response
	GRIK4	rs1952787 T/C	261	0.500	normal response
		rs1952787 C/C	155	0.297	normal response
		rs1952787 T/T	106	0.203	risk of reduced response
	HTR2A	rs7997012 A/G	265	0.508	intron 2 genotype AG
		rs7997012 G/G	147	0.282	intron 2 genotype GG
		rs7997012 A/A	110	0.211	intron 2 genotype AA
	HTR2C	rs3813929 C/C	380	0.728	normal risk
		rs3813929 C/T	88	0.169	protective effect
		rs3813929 T/T	54	0.103	protective effect
	OPRM1	rs1799971 A/A	399	0.764	Asn/Asn isoform
		rs1799971 A/G	116	0.222	Asn/Asp isoform
		rs1799971 G/G	7	0.013	Asp/Asp isoform
**OTHER**	HLA-A	Negative	503	0.964	normal risk
		Positive *31:01	19	0.036	increased risk
	HLA-B	Negative	485	0.929	normal risk
		Positive *57:01	20	0.038	increased risk with abacavir and pazopanib
		Positive *58:01	17	0.033	increased risk with allopurinol
	IFNL4	rs12979860 C/C	248	0.475	normal response
		rs12979860 C/T	219	0.420	reduced response
		rs12979860 T/T	55	0.105	reduced response
	SLC6A4	La/Sa	198	0.394	typical to reduced expression
		La/La	151	0.300	typical to increased expression
		Sa/Sa	84	0.167	reduced expression
		La/Lg	44	0.087	likely typical to reduced expression
		Lg/Sa	26	0.052	likely reduced expression
		La/Sa	198	0.394	typical to reduced expression
	SLCO1B1	*1/*21	7	0.013	reduced response
		*1/*17 OR *5/*21	10	0.019	increased risk
		*1A/*1A	84	0.161	normal risk
		*1A/*1B	122	0.234	normal risk
		*1A/*5	28	0.054	increased risk
		*1A/*21	28	0.054	reduced response
		*1A/*15 OR *1B/*5	80	0.153	increased risk
		*1A/*17 OR *5/*21	17	0.033	increased risk
		*1B/*1B	94	0.180	normal risk
		*1B/*15	27	0.052	increased risk
		*1B/*21	2	0.004	reduced response
		*1B/*17 OR *15/*21	8	0.015	decreased function
		*5/*5	3	0.006	increased risk
		*5/*15	2	0.004	increased risk
		*5/*17	1	0.002	increased risk
		*15/*15	5	0.010	poor function
		*17/*17	1	0.002	increased risk
		*17/*21	2	0.004	increased risk
		*21/*21	1	0.002	reduced response
	F II	rs1799963 G/A	16	0.031	increased risk
		rs1799963 G/G	506	0.969	normal risk
	F V	rs6025 G/A	17	0.033	increased risk
		rs6025 G/G	505	0.967	normal risk

## Data Availability

All of the research data is included in the manuscript.
